# The informative value of museum collections for ecology and conservation: A comparison with target sampling in the Brazilian Atlantic forest

**DOI:** 10.1371/journal.pone.0205710

**Published:** 2018-11-14

**Authors:** Vitor Dias Tarli, Philippe Grandcolas, Roseli Pellens

**Affiliations:** 1 Institut de Systématique, Evolution, Biodiversité (ISYEB—UMR 7205)–Muséum national d’Histoire naturelle, CNRS, Ecole Pratique de Hautes Etudes, Sorbonne Université –CP50, Paris, France; 2 CAPES Foundation–Ministry of Education of Brazil, Brasilia, DF, Brazil; National Institute of Biology, SLOVENIA

## Abstract

Since two decades the richness and potential of natural history collections (NHC) were rediscovered and emphasized, promoting a revolution in the access on data of species occurrence, and fostering the development of several disciplines. Nevertheless, due to their inherent erratic nature, NHC data are plagued by several biases. Understanding these biases is a major issue, particularly because ecological niche models (ENMs) are based on the assumption that data are not biased. Based on it, a recent body of research have focused on searching adequate methods for dealing with biased data and proposed the use of filters in geographical and environmental space. Although the strength of filtering in environmental space has been shown with virtual species, nothing has yet been tested with a real dataset including field validation. In order to contribute to this task, we explore this issue by comparing a dataset from NHC to a recent targeted sampling of the cockroach genus *Monastria* Saussure, 1864 in the Brazilian Atlantic forest. We showed that, despite strong similarities, the area modeled with NHC data was much smaller. These differences were due to strong climate biases, which increased model’s specificity and reduced sensitivity. By applying two forms of rarefaction in the environmental space, we showed that deleting points at random in the most biased climate class is a powerful way for increasing model’s sensitivity, so making predictions more suitable to the reality.

## Introduction

Natural History Collections (NHCs) were designed to keep vouchers of the living world several centuries ago. More than a simple repository for taxonomic studies, these collections are memories of the past and present life on earth, and represent important references of biodiversity in time and space. In the last two decades, the richness and the huge potential of these collections have been rediscovered and emphasized [1–3]). Many possible uses have been listed for specimens housed in collections [4, 5]), as for example, tracking invasions [6]), defining trends in populations of pathogens and parasites [7]), revealing the history of diseases [8, 9]), analyzing responses to environmental changes [10, 11], building seed banks [6]), following phenotypic and genotypic changes in populations and documenting many aspects of the evolutionary process [12].

This recent emphasis on NHC data also brings lots of benefits for studies of macroecology. The international enterprise of rendering available data from specimen’s labels (and associated information from field notes and expedition logs), and more recently, traits and pictures of the specimens, is powering this research field, which is becoming central in ecology and biodiversity conservation [13]. The massive amount of data available in national databases and some data federators like GBIF (http://www.gbif.org) along with environmental data interpolated at high spatial resolution (e.g. [14, 15]) and powerful methods of analysis does not only allow for unraveling main patterns of biodiversity distribution, but also for understanding the processes leading to them (see [13] for a review).

However, most of the specimens housed in collections were not necessarily collected based on protocols and standardized samplings. Most of them come from the accumulation of erratic field works over more than two centuries. Assembling them to answer a specific question requires considering the biases that they may span. For example, the well-known biases towards places of easy access [near waterways, roads (e.g. [16, 17])], in areas with high population density (e.g. [18] for Europe, but see [19] for China), with good academic [20, 21], or socio-economic structure [22]]; and biases away from remote regions (e.g. [23]).

Depending on the constraints of access, and on the regional environmental variability, these biases might have important implications on the environmental range sampled [17, 24], and on the inferences of species’ distribution range (e.g. [23, 25]. This makes that the use of NHC data is very challenging, particularly because ENMs as estimated in from presence-only models [26] are based on the assumption that distribution records are not biased [27]. Due to this, a whole body of research has been devoted to the characterization of biases in collection databases and to the search of solutions in order to minimize errors on estimates based on ENMs [28, 29]. However, the lack of field validation still represents a major constraint for evaluating and understanding models’ outcomes ([10, 30]). Field data is very necessary for confirming distribution, assessing eventual biases in the samples from NHC, so allowing to go a step further and developing solutions for using them in biodiversity assessments.

During a biogeographic study in the Brazilian Atlantic forest, we took advantage of a long-term survey of the insect genus *Monastria* Saussure, 1864 (Dictyoptera, Blaberidae) to mobilize data for this kind of study. We referred to all Museum collections in the world that harbored specimens of *Monastria* and we conducted a field sampling designed to characterize their distribution in the biome and to define the limits of their distribution range. The main interest of focusing on species of this saprophagous genus is that they are not specialized, so not constrained by specific resources like a host plant [31, 32, 33]). They typically represent that important fraction of biodiversity that is actually not well-known or even followed on a regular basis, contrarily to some vertebrates, and therefore necessitates that all available data are mobilized for its study [34].

Here we used all distribution records available to the species of this genus aiming to explore whether data issuing from NHC dataset would be enough to predict its entire distribution range, as validated by the recent sampling dataset. Based on it, we explored how sampling biases could be responsible for the result. Then, we developed two strategies of rarefaction and compared the way they influenced the outcomes of ENMs. The study was made in the Brazilian Atlantic forest, a diverse forest ecosystem, comprising several different physiognomies. Our main expectation is that the comparison of samples from NHC with present sampling will unravel trends commonly found in NHC datasets, so allowing to explore what leads to them, and some ways to deal with them if we aim to produce sound biodiversity assessments.

## Material and methods

### The study model

Cockroaches of the genus *Monastria* belong to the Neotropical subfamily Blaberinae [35–37]. The genus includes nine species. Three of them with large and partially overlapping distribution range, and six others known from single isolated localities [33]). Species of this genus are historically known from the Brazilian Atlantic Forest [38], occurring from the State of Ceará to the Rio Grande do Sul in the South of Brazil (03° to 30°S), and from the Atlantic coast to the furthest inland forests of this biogeographical domain, in Misiones (Argentina) and in Assumption (Paraguay). They were observed in a large array of ecosystems composing this biome, ranging from semi-deciduous forests in the Northeast to the humid montane forests in the central region and the *Araucaria* forests in the South. Individuals of *Monastria* shelter on the underside of dead trunks lying on the forest ground, have a generation time of about 2 years, are very sedentary and gregarious, and adults reach the size of small vertebrates (about 3cm in length x 1.5cm in width). They are collected by direct search on their specific habitats, or, indirectly, by collectors searching for xylophagous insects. Adult males can be captured with light traps, although it rarely occurs [31,32, 39].

### Collection data

We searched for *Monastria* in collections of Natural History Museums (NHM) and in the literature. The survey in NHM was made through contacts and specific requests to the curators of the main repositories of Neotropical fauna in the world. This was very often complemented by exchanges of pictures in order to specify the cockroaches we were looking for. Concerning the literature, we relied on the catalogue of [40], and the updates available on the Taxonomic Catalogue of the Brazilian Fauna at http://fauna.jbrj.gov.br/fauna/listaBrasil/ConsultaPublicaUC/ResultadoDaConsultaNovaConsulta.do, which provides an exhaustive and updated survey of the publications on the Blattaria from Brazil. This led to a dataset issuing from 23 references (S1 Appendix) and 11 collections (S1 Table). We assigned geographical coordinates to every specimen with enough information at the level of a locality or with more details. Specimens with information of occurrence at very coarse resolution (level of the continent, a country, a state, or a big city) were discarded.

### Target sampling

We designed a sampling protocol aimed at checking the occurrence in different forest physiognomy within the Atlantic Forest and at characterizing longitudinal, latitudinal and altitudinal limits of distribution. Since the Atlantic forest is now reduced to less than 5% of its original surface and distributed in a multitude of scattered fragments [41], we focused mainly on officially protected areas. But some forests in private properties in regions where reserves do not exist were also sampled. Based in a first study, in which we verified that individuals of *Monastria* were not present in tree plantations, or secondary regrowth forests, even when they were very near forests where they were abundant (i.e. less than 1km) [32], we limited our fieldwork to forests. The main requirement was that each forest site prospected had at least three strata, as well as dead trunks and branches in the understory. Every forest physiognomy of the biome and all forests located at the extreme of distribution of the Atlantic forest were sampled. This made a total of 26 sites with presence and 21 with absences.

In each forest, sampling was made through walks perpendicular to main trails looking for their microhabitat, i.e., dead trunks lying in the forest ground. Each trunk observed was turned in order to search for individuals. This procedure was repeated until finding at least one individual. Absences were assumed after 8 hours of field search, period in which at least 20 clumps of dead trunks were prospected. In represents search in about 4ha, or along at least 5km of trails. The great majority of the absences recorded here are related to the present quality of site, i.e. in some regions the only forest remaining are either very disturbed native forests or secondary old regrowth. This environment markedly reduces the chances of finding *Monastria*. For this reason, the absences were not included in the models.

### Climate data

We used Bioclim variables obtained in WORLDCLIM Version 1.4 database (http://www.worldclim.org; [14]), in 30-arc second resolution, or about 1km x 1 km near the equator. In order to reduce collinearity (e.g. [28]), we eliminated variables where Pearson’s r >0,80 and retained the ones correlated with more variables. So, the analysis was limited to only eight of them (Table 1).

**Table 1:** The eight bioclim variables used in this study. Abbreviation, full name, minimum and maximum values of the occurrence records from the target sampling (TS), and natural history collections and literature (NHC) dataset. The last columns present the difference between the two datasets and the sum of these differences.

Abbreviation	Variable	TS		NHC		TS—NHC		
		Min	Max	Min	Max	Min	Max	SUMM
bio01	Annual Mean Temperature	154	242	152	255	2	-13	-11
bio02	Mean Diurnal Range^a^	63	130	64	140	-1	-10	-11
bio03	Isothermality^b^	46	69	47	67	-1	2	1
bio05	Max Temperature of Warmest Month	233	321	248	338	-15	-17	-32
bio12	Annual Precipitation	1197	2102	1177	2171	20	-69	-49
bio13	Precipitation of Wettest Month	173	313	132	338	41	-25	16
bio14	Precipitation of Driest Month	11	124	8	156	3	-32	-29
bio15	Precipitation Seasonality^c^	10	81	9	86	1	-5	-4

Temperature values are given in°C*10, precipitation in mm.

^a^ Mean of monthly (max temp—min temp)

^**b**^ (mean diurnal range/annual range) (*100)

^**c**^ Coefficient of Variation of monthly precipitation).

### Analysis

ENMs were modeled with MaxEnt 3.3.3 [26]. We chose to use this method due to its excellent predictive performance when compared to several other ENM methods, independently if they are based on presence only or if they characterize background with a sample [42–44]. In all analyses performed in this study, 70% of the data was used in training and 30% was retained as test points. We employed the subsample parameter for the replicates and set “maximum training sensitivity plus specificity” as the threshold, which means that habitats are labeled as suitable when probability ≥ threshold. The parameters for the maximum number of interactions and replicates were set as 5000 and 20, respectively, and all analyses were based on the mean of the 20 replicates. MaxEnt predictions are presented in a continuous cumulative probability field. We transformed this probability field into binary maps of “suitable” (upper class) versus “unsuitable” for calculating and comparing the distribution area. These maps were transformed into polygons used to calculate the final area with ArcGis 10.4. The Area Under the Curve (AUC) on Receiver Operating Characteristis (ROC) plots of training and test was used to validate the models. In order to avoid problems in comparisons of these estimators the geographic extent of the models was always the same [45].

The similarity between the two ENM’s was quantified with the I-statistics using the program ENMTools [46]. This statistic compares the overlap of full grid-cells in a given area, producing results varying from 0 (no overlap) to 1 (identical models).

### Assessing biases and analyzing its effect in the dataset

The distribution of sampling points in the dataset was assessed in two ways. The first was the estimation of the aggregation of points in the geographic space. It was tested with Averaged Nearest Neighbor calculated in ArcGis 10.4. This test verifies if distances between nearest neighbors are different than what would be expected if they were at random. The second was the evaluation of sample aggregation in climate space, i.e., if samples were aggregated in places having a certain type of climate in common even when these places were scattered apart geographically. This was done through the assessment of differences in probability of occurrence between observed and expected number of points ([17, 24]. Following the basic MaxEnt output, the climate space was divided into 9 equal-interval bins based on the range observed within the Atlantic Forest. For instance, the interval between maximum and minimum values of each climate variable was divided in 9 classes, each comprising 1/9^th^ of the values, and calculated the area covered by each class. Then we calculated the number of sampling points and the proportion of points expected based on the area covered by each bin. This was based on the expectation that if samples were not biased, they would correspond to the proportion represented by that climate space in the total. For each climate variable, bias was calculated as:

$${Bias}_{\begin{array}{c} d \end{array}}=\frac{n_{d}-p_{d}N}{\sqrt{p_{d}(1-p_{d})N}}$$

where n_d_ is the number of localities collected within climate bin_d_, p_d_ is the probability that a collecting locality falls within climate bin d given the area covered by that bin, and N is the total number of collecting localities. In other words, this formula compares the number of samples observed with that expected, assuming that the probability of being collected in a fraction of the climate is proportional to the total area comprised by it.

In order to check the implications of climate biases on the ENMs of collection data we designed a rarefaction strategy to delete points in order to make subsets of the dataset. We limited this analysis to Annual Precipitation based on the fact that this variable is the one with greatest difference in range covered between the niches with the two datasets. Two forms of rarefaction were employed. In the first we eliminated 30%, 40%, 45% and 55% of the points from the most skewed climate class (11, 15, 17 and 21 points, respectively) chosen at random. In the second, we deleted the same number of points at random from the entire dataset. Comparisons were made with results of twenty replicates for each situation. A One-way ANOVA (single factor) was used to compare the effect of rarefaction on the AUC training, test and area values. A two-way ANOVA (two factors) was employed to compare the effect of two ways of rarefaction (deleting at random from the entire set, or deleting at random on the most biased class) and of number of points deleted (11, 15, 17, 21).

## Results

### Characterization of the datasets

**Fig 1:**
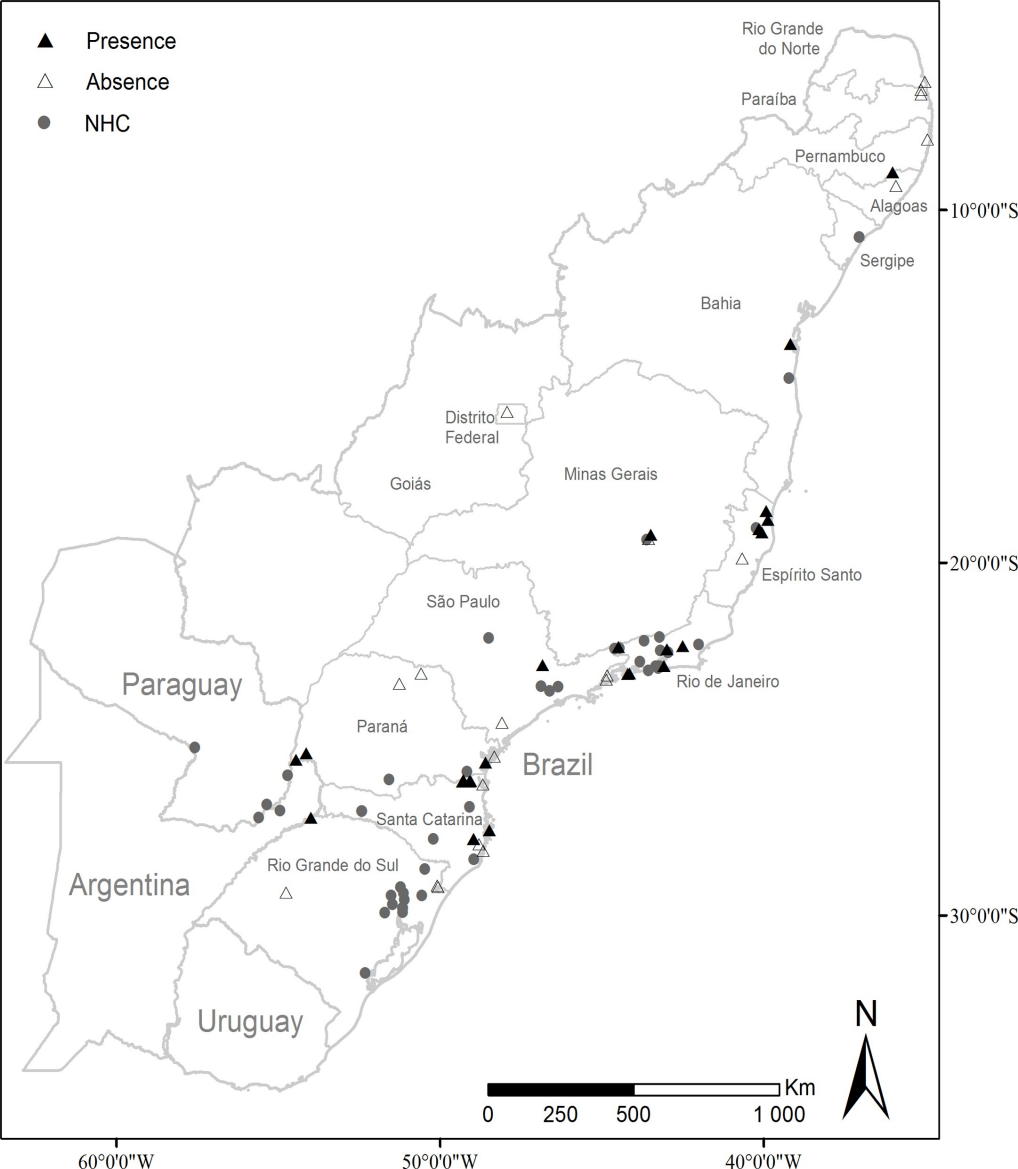
Distribution of the sampling records of *Monastria* in the Brazilian Atlantic forest. Data from NHC: full circle; Data from TS: presence (full triangle), absence (empty triangle).

Our dataset was composed of 82 occurrence data: 56 from Museum collections and literature (hereafter NHC) resulting from 23 independent samples (S1 Appendix), and 26 from the called target sampling (TS). Twenty-one additional locations were studied with the target sampling without finding *Monastria*. As most of these absences looked associated to the present forest degradation, they were not used as pseudo-absences. Both occurrence records cover about the entire range of the Atlantic forest. But NHC dataset includes records much further in the South and West whereas the TS dataset includes presences in the extreme Northeast (Fig 1). Despite these differences in the geographical space, the range of the occurrence in environmental space is quite similar. As can be seen by the sum of the differences between minimum and maximum values with the two datasets, annual precipitation is the variable with the highest difference of range (Table 1).

### Assessing distribution with the two different datasets

**Fig 2:**
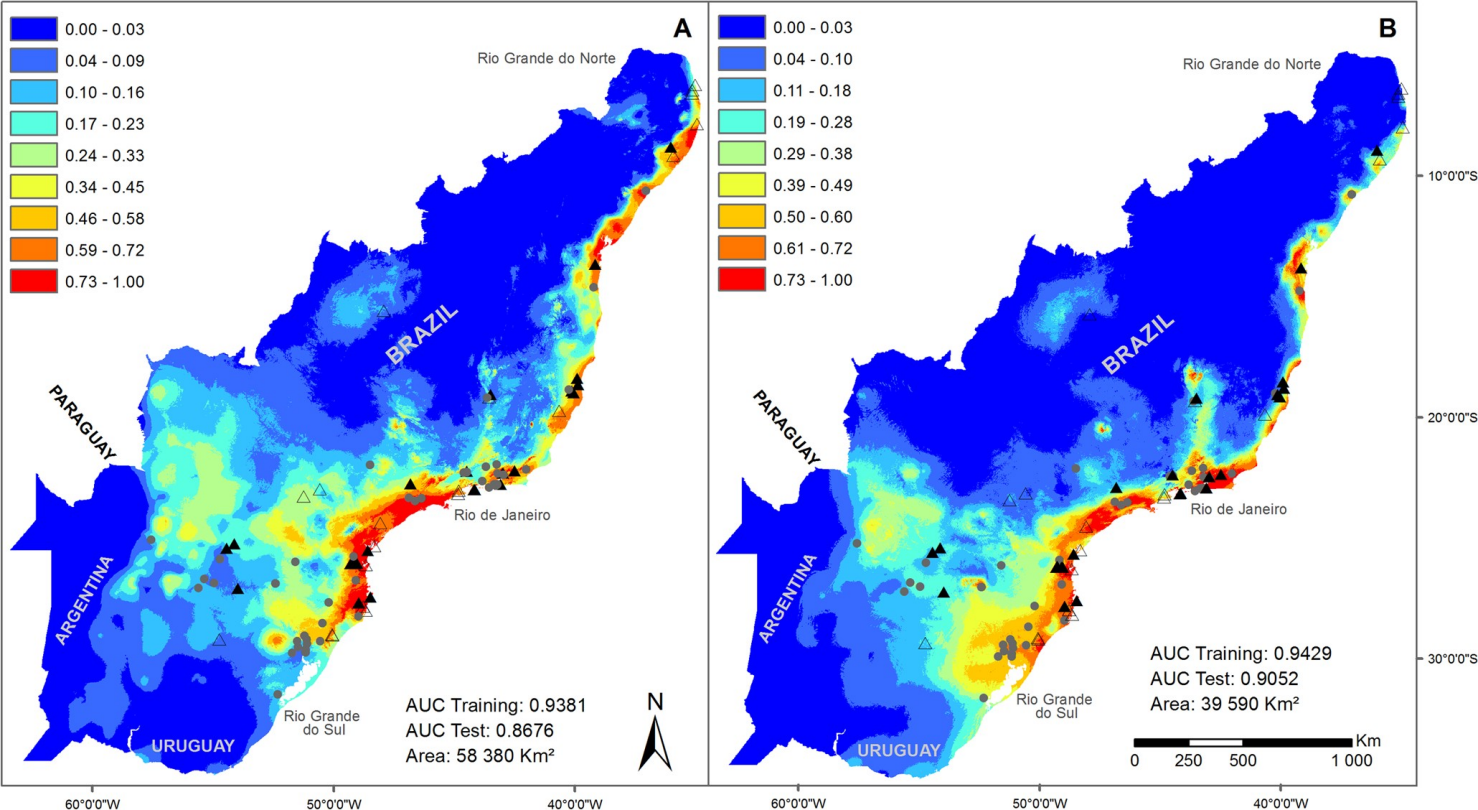
Ecological niche models of the cockroach *Monastria* in the Neotropical Atlantic forest. Ecological niche of the cockroach *Monastria* in the Neotropical Atlantic Forest modeled with two different datasets. A) Data from TS; B) Data from NHC. Values of AUC training, test and area are the mean of 20 replicates.

MaxEnt performed well in both analyses. The training AUC (area under the receiver operating characteristic curve) was slightly higher for the ENM with collection data (0.9429), than in the ENM with data from the target sampling (0.9381). In both cases it strongly rejected the hypothesis that test points were predicted no better than by a random prediction. No locality point fell outside the total distribution area predicted by the model, although some of them were found in areas with low predicted suitability. The I-statistics indicates that the entire area of ENMs estimated with the two datasets strongly overlap (I = 0.92) (Fig 2).

The analysis of contribution of the different variables indicated that Bio02 was the one with highest regularized trained gain, with 31.1% and 29.2%, followed by Bio03 and Bio14 for collection and target sampling, respectively. It shows that the most suitable areas for *Monastria* were those with low mean diurnal range in temperature (Bio02 and Bio03), which, in this region, was mainly determined by variations in precipitation during the driest month (Bio 14) (Table 2).

**Table 2:** Relative contributions and permutation importance of the variables used for modeling the niche of *Monastria* with data issuing from two different datasets.

		TS		NHC	
	Variable	Percent contribution	Permutation importance	Percent contribution	Permutation importance
bio01	Annual Mean Temp	0.2	0.2	0.7	1.2
bio02	Mean Diurnal Range	29.2	20.4	31.1	25.2
bio03	Isothermality	1.4	7.6	24.5	48.2
bio05	Max Temp Warmest Month	16.7	6.5	8.6	2.1
bio12	Annual Precipitation	0.5	0.1	12.9	16
bio13	Precip of Wettest Month	20.1	33.8	2.7	0.8
bio14	Precip of Driest Month	27	27.9	18.8	1.9
bio15	Precip Seasonality	4.9	3.6	0.7	4.6

**Fig 3:**
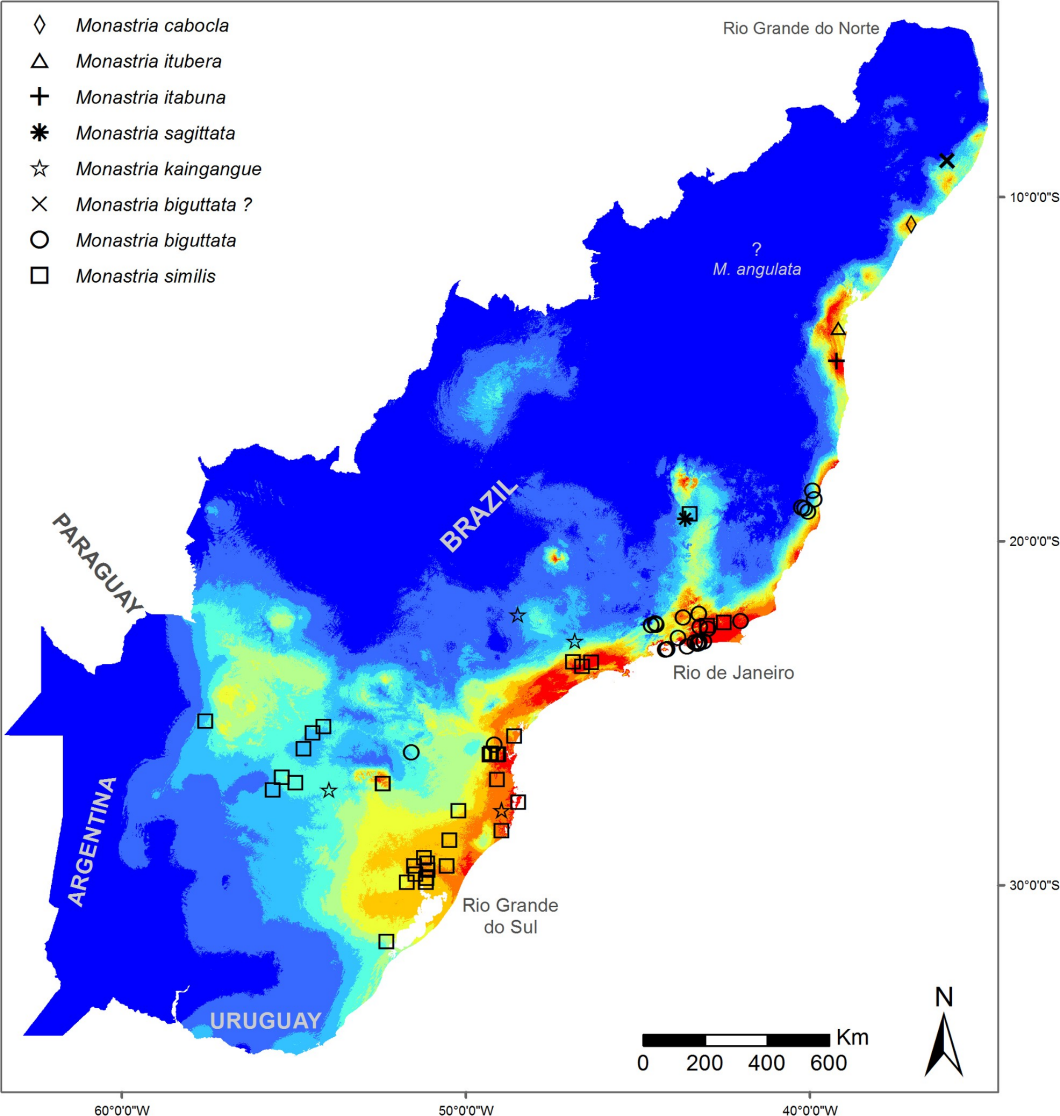
Distribution of the nine species of *Monastria* in the ENM’s dataset from NHC. According to the article 8.2 and 8.3 of the International Code of Zoological Nomenclature, the present publication is not issued for the purposes of zoological nomenclature and the names or acts displayed are not available and disclaimed.

**Fig 4:**
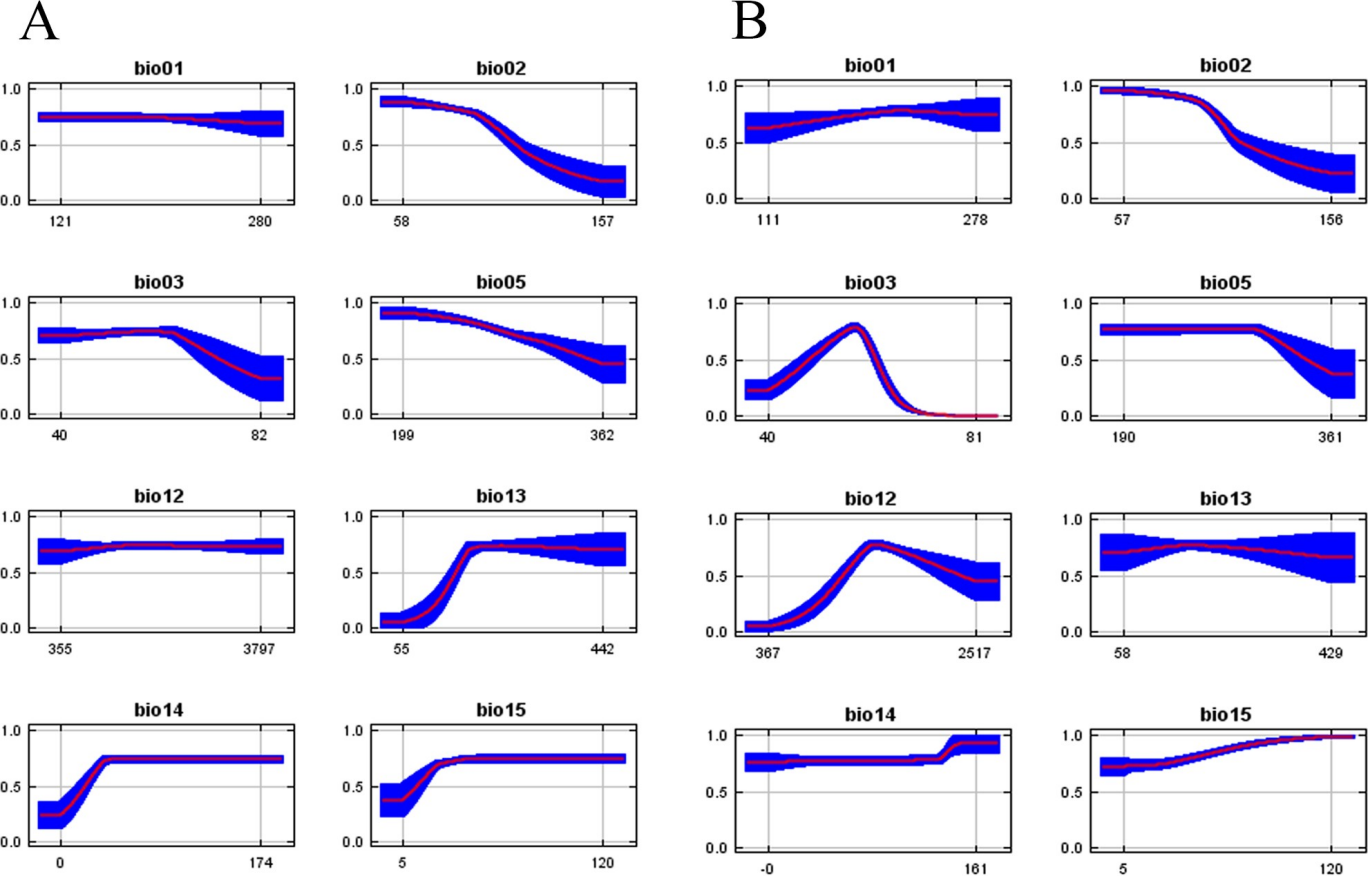
The response curves of the eight bioclim variables used in this study. The curves show the mean response of the 20 replicate MaxEnt runs (red) and the mean +/- one standard deviation (blue).

In spite of this, the ENMs differed markedly in extent of suitable area. The range estimated with NHC data corresponded to only 67% of that with our recent sampling, indicating suitable areas much concentrated in the humid forests at the central region of the biome, particularly in the region of Rio de Janeiro. The model produced with the TS dataset showed additional suitable areas in the Northeast, where *Monastria* was not known before. Another important difference was detected in the extreme South at the interior of Rio Grande do Sul, both with several records in the NHC dataset, but not identified as suitable with the model produced with it (Fig 2). As a result of this failure to detect suitable areas at the extreme Northeast, the range of two out of nine species of this genus were not or were very poorly detected with the dataset from NHC (Fig 3). The response curves show that annual precipitation (Bio12) was the environmental variable with highest difference between the two models, with a range about 1/3 wider in the models with data from the target sampling (Fig 4).

### Assessing biases in the datasets

The test of spatial aggregation showed that, although values were significant for both datasets, they were much higher in the data from NHC (Z-score = -5,892; p< 0,0001) than that in the target sampling (Z-score = -2,2901; p = 0,022). It means that the observed average distance between points was much lower than expected at random, especially in the NHC dataset.

The analysis of climatic biases shows that the intermediate climate class 4 was the most sampled in both datasets. Nevertheless, biases were much higher (more than twofold) with data from NHC than with data from the TS dataset, particularly for Bio2, Bio5 and Bio12 (Table 3).

**Table 3:** Values of bias_d_ calculated with data from a target sampling (TS) and data from natural history collections and literature (NHC) for eight climatic variables used to estimate ENMs of *Monastria* in the Brazilian Atlantic forest. Highest values are indicated in bold.

	Bio01		Bio02		Bio03		Bio05		Bio12		Bio13		Bio14		Bio15	
	Mean Annual Temperature		Mean Diurnal Range in Temp		Isothermality		Max Temp of Warmest Month		Annual Precipitation		Precipitation of Wettest Month		Precipitation of Driest Month		Precipitation Seasonality	
Climate classes	TS	NHC	TS	NHC	TS	NHC	TS	NHC	TS	NHC	TS	NHC	TS	NHC	TS	NHC
1	-2.17	-2.08	-0.61	0.00	0.00	3.56	2.46	-1.62	-1.02	-2.34	-1.02	-1.44	-1.63	-0.94	-1.02	4.04
2	-3.40	-2.67	0.00	-0.52	2.04	2.88	1.47	2.37	-1.84	-2.59	-2.17	0.40	0.74	0.36	-1.23	0.43
3	1.00	0.00	1.84	1.30	1.47	0.00	-0.61	0.00	1.47	0.00	0.00	0.00	2.46	0.38	-1.63	-1.82
4	-2.42	0.70	**5.15**	**9.34**	1.47	1.82	**0.00**	**6.09**	**4.35**	**9.77**	0.00	0.00	-1.09	-0.40	5.44	-2.18
5	-1.40	1.73	-1.09	-2.18	1.02	1.91	-1.23	-0.93	3.07	0.86	-0.74	-0.34	3.68	-0.40	3.68	1.78
6	-0.93	1.73	-1.00	0.34	-0.54	-2.29	-1.47	1.19	-1.49	0.52	2.17	-0.43	1.47	2.02	-1.63	1.30
7	-1.40	0.86	-1.40	-1.73	0.47	0.52	1.63	-1.82	-1.84	-3.06	-0.74	1.19	-0.61	0.52	-1.02	-1.62
8	-0.47	0.43	-1.02	-3.14	-2.79	-3.91	-1.23	-2.42	-2.17	-2.34	0.74	1.73	-2.49	-2.42	-1.09	1.78
9	-2.49	-0.94	-1.02	-1.44	-0.74	-1.78	-1.47	-0.52	-1.02	-2.83	1.23	-1.15	-1.02	1.44	-1.84	-2.16

### Effect of rarefaction on the collection dataset

Since Bio12 was the environmental variable with highest difference in range between the two models (Fig 4) we chose to use it to test the effect of rarefaction on the environmental space.

As expected, AUC values were significantly reduced with rarefied data, especially AUC training (One-way ANOVA F = 4.4185 *p*<0.0001 DF = 8) but also for AUC test (F = 2.9906; *p =* 0.004; DF = 8). But, the estimated suitable areas were significantly higher (F = 11.72348 *p*<0.0001 DF = 8).

**Fig 5:**
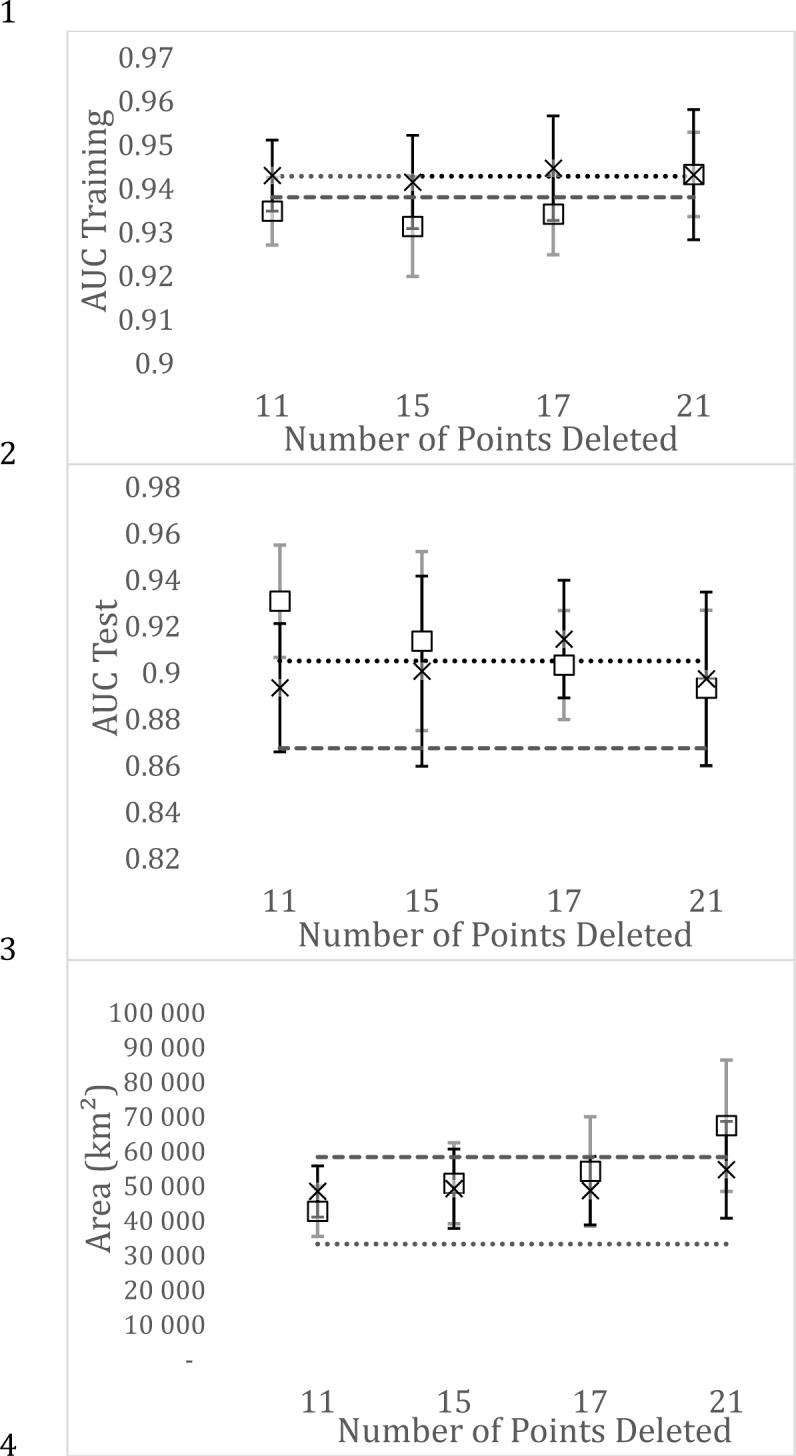
AUC training, AUC test and area estimated with NHC and literature data rarefied in two different ways. ϒ Mean and SD (gray line) using a dataset in which points were deleted at random only from the most biased climate class of Annual Precipitation (class 4 in Table 2); × Mean and SD (black line) using a dataset in which points were deleted at random in the entire dataset. In both cases the same number of points was deleted. They represented 30, 40, 45 and 55% of the points in the most biased climate class. Dotted line: Mean values estimated with the entire dataset from NHC. Dashed Line: Mean values estimated with the entire dataset from TS.

The comparison of two ways of rarefaction showed important differences concerning AUC training and area. AUC training varied markedly and not linearly when the dataset was rarefied by deleting points in the most biased climate class. But, when 21 points was deleted, the values from the two modes of rarefaction were very similar and also similar to the that estimated with all the NHC dataset. The values of AUC test strongly varied among the 20 models produced for each situation, as shown by the higher standard deviation (bars in Fig 5), so showing no significant differences between ways of rarefaction, except for the interaction (Table 4). Concerning suitable area, the differences between the two ways of rarefying increased with the number of points deleted. In the class with 21 points (55%) deleted, the area estimated with data rarefied in the most biased climate class was even broader than that obtained with target sampling (Fig 5; Table 4).

**Table 4:** Results of two-way ANOVA comparing the effect of rarefaction on the collection data (See Fig 2 for more information).

	Mean Square	d.f.	*F*-value	Significance
AUC Training				
Entire dataset X Most Biased dataset	0.002	1	18.0288	**< 0.0001**
Number of Points Deleted	0.0003	3	2.7477	**0.0449**
Interaction	0.0002	3	2.1301	0.0987
AUC Test				
Entire dataset X Most Biased dataset	0.003	1	2.9773	0.0864
Number of Points Deleted	0.0021	3	2.0755	0.1058
Interaction	0.0046	3	4.5134	**0.0046**
Area				
Entire dataset X Most Biased dataset	500478428	1	3.1422	0.0782
Number of Points Deleted	1700097789	3	10.674	**< 0.0001**
Interaction	593681737	3	3.7274	**0.0127**

Bold numbers correspond to a statistical significance (p <0.05)

## Discussion

Niche models obtained with NHC or with TS had particularly high performance, especially because of the important breadth of the distribution range (Fig 4) [47]. Nonetheless, as validated by the sampling records, the prediction with the TS dataset were more adjusted to the real distribution of *Monastria* in its entire range. Had we used the model with the NHC dataset to predict where to find new species of *Monastria*, two species would have been unnoticed. The differences between the predictions made with the two datasets were not only in regions under-sampled by the NHC collection dataset, as in the Northeast, but also in regions well sampled in the South and Southwest. This suggested that the problem was not in the geographic, but in the environmental space.

This hypothesis was confirmed by the analysis of climate biases, which showed significant differences in representation in different climate bins between the two datasets. Biases in sampling arise by (1) overrepresentation of samples in some climate classes (positive values), (2) absence or low representation in others (negative), or (3) a combination of both. Here we identified that collection data of *Monastria* were strongly overrepresented in moderate climate ranges. At least two main and non-exclusive hypotheses can be raised to explain this result. The first is that the number of samples reflects the abundance, so indicating the optimum environments to *Monastria*, which would lead to higher probability of being collected. A second hypothesis is that the places in these climates are the ones more frequently visited by researchers and collectors in general. So, the number of samples reflect facility of access or site attraction. A study of the sampling effort of several groups of organisms in the same region could help to verify this tendency.

The results of the rarefaction confirmed the conclusions on the importance of sampling biases for explaining the differences in area in ENMs estimated with the two datasets. The increase in estimated suitable area with rarefaction independently of the way data were deleted brought one more argument to the importance of filtering. Some studies have shown that suitable areas also increased when filtered in geographical space [28, 29], i.e. by deleting redundant points occurring at an arbitrary distance from each other. However, a recent study comparing the effects of filtering in geographical and environmental space for virtual species showed that the utility of geographic filters was quite unlikely to be generalized to several places. In fact, it could increase climate biases in areas with heterogeneous and repeated environments across different geographic scales [48].

The second point contributing to this conclusion was that rarefaction did not necessarily imply a decrease in model performance, as shown by variations in AUC. This was contrary to that observed by [28, 48] when using spatial filters, and in accordance with the observation of [48] when using environmental filter. It indicated that, when environmental bias was reduced, other combination of variables became evident, so leading to robust models with much less data (Fig 5).

Although debiasing is an important issue, excluding data is a crucial choice when dealing with NHC datasets [26], particularly because very often the number of data available is not enough to make good inferences on the species distribution range [25]. Nevertheless, as shown by the present results, and also by [28, 29, 48, 49], if biases are detected it is necessary to find a way to reduce it, otherwise it will mask the reality of the distribution range.

Our results emphasize that testing for climate biases [17, 24] is a very important step in this evaluation. They show that overrepresentation of samples in a climate class favor the maximization of model’s specificity. This means that the suitable areas are predicted in climate spaces with higher number of records. In other words, the model outcomes are very good at finding true positives, but it fails in predicting some false negatives, i.e. it predicts the absence in some places where the species really occurs.

The second outcome of this study is how to filter in order to enhance model’s sensitivity. By comparing two strategies of deleting points at random in the environmental space, we showed that acting on the most biased climate class is more effective, which allows to detect other suitable areas.

This calls the attention to the importance of clearly defining the aim of the study when using SDMs in order to decide the best way to use the data available [50]. For example, if we are looking for the best site to place a reserve, it is desirable to maximize specificity (i.e. the chances that the species occur in the site). So, considering all points may be the good choice, as it reduces the chances of commission errors, i.e. the probability of inferring the presence when a species is not there. It implies in avoiding errors in estimates of species richness, for example, which would lead to the creation of reserves when species are not really confirmed to be there [51]. Nonetheless, if the aim is to screen all possible habitats in order to find new species of the same genus as in our study, or to make inferences about future availability of suitable habitats, sensitivity is highly important. In this case, detecting environmental biases and rarefying by reducing the number of occurrences on the most biased classes can be a worthful strategy, as it leads to robust models enlarging the possibility of places to be screened.

A final point to be considered concerns the use of a genus (even if having a small number of species) whereas ENMs are designed for working at species level. Theoretically, the main reason for working at species level is the assumption that all populations of a same species would have similar mean environmental optima with variances at least partly overlapping. More studies are necessary to understand the mode of evolution of the niches of *Monastria*, in order to understand if the results found fit a theoretical case in which niches evolved “randomly” or not. In the first case, it would be perfectly fitting the assumptions for using ENMs at the level of a genus. If not, it would indicate some other cases in which the use of ENMs at genus level is worth to apply. However, the results of this analysis indicate strong possibility of making good inferences for the occurrence of all species in the dataset, even in cases for which very few points are available. This makes that the use of ENMs at the genus level opens to the possibility of inferring where other species in a clade may be found.

To conclude, NHC is a goldmine of data readily available to be used in biodiversity science. But, as these data do not become from a pre-defined sampling protocol to answer a specific question, studying how samples are distributed and detecting possible biases is very necessary. In this respect, field validation is crucial, as it is the only way to test the predictions [10, 30]. The study of genus *Monastria* in the Brazilian Atlantic forest showed the need to look for climate biases in SDM, and the solution proposed here is likely to be useful in any situation in which overrepresentation of samples in a climate class is detected.

## Supporting information

S1 Appendix Exhaustive list of references with location records used in NHC dataset. (DOCX)

S1 Table List of natural history collections with specimens of *Monastria*. (DOCX)
