# Supplementary material for: The informative value of museum collections for ecology and conservation: A comparison with target sampling in the Brazilian Atlantic forest
Source: PLoS One. 2018 Nov 14;13(11):e0205710. doi: 10.1371/journal.pone.0205710 (PMC6235285; doi:10.1371/journal.pone.0205710)
Supplement: S1 Appendix — (DOCX) [file pone.0205710.s001.docx]

**S1 Appendix. Exhaustive list of references with location records used in NHC dataset.**

1. Becker, C. J. 1984. Preliminary survey of the Blattaria of the state of Rio Grande do Sul, Brazil. Revista Brasileira de Entomologia. 28(1):87-98.
2. Brunner von Wattenwyl, C. (1865) *Nouveau Système des Blattaires*. Vienna, Austria, 426 pp.
3. Couri, M., Nessimian, J., Mejdalani, G., Monne, M.L., Fraga, S.M.L., Mendonca, M.C., Monteiro, R.F., Buys, S.C. & Caravalho, R. A. 2009. Levantamento dos insetos da Mata Atlântica do Estado do Rio de Janeiro. *Arquivos do Museu Nacional do Rio de Janeiro*, 67:151-154.
4. Hebard, M. (1921) South American Blattidae from the Museum National d'Histoire Naturelle, Paris, France. *Proceedings of the Academy of Natural Sciences of Philadelphia*, 73, 193−304.
5. Pellens, R. & Grandcolas, P. (2003) Living in Atlantic forest fragments: life habits, behaviour and colony structure of the cockroach *Monastria biguttata* (Dictyoptera, Blaberidae, Blaberinae) in Espirito Santo, Brazil. *Canadian Journal of Zoology*, 82, 1929–1937.
6. Pellens, R. & Grandcolas, P. (2007) The conservation refugium value of small and disturbed Brazilian Atlantic forest fragments for the endemic ovoviviparous cockroach *Monastria biguttata* (Insecta: Dictyoptera, Blaberidae, Blaberinae). *Zoological Science*, 24, 11–19.
7. Pellens, R. & Grandcolas P. (2008) Catalogue of Blattaria (Insecta) from Brazil. *Zootaxa* 1709, 1–109.
8. Princis, K. (1946) Colombianische Blattodeen, gesammelt von Herrn G. Dahl und Frau M. Althin-Dahl in den Jahren 1936–1939. *Kungl. Fysiografiska Sallskapets I Lund Förhandlingar*, 16, 162 pp.
9. Princis, K. (1963) Blattariae: Suborde Polyphagoidea: Fam.: Homoeogamiidae, Euthyrrhaphidae, Latindiidae, Anacompsidae, Atticolidae, Attaphilidae; Subordo Blaberoidea: Fam. Blaberidae. *In*: Beier M (Ed.) Orthopterorum Catalogus*,* Pars 4. Uitgeverij Dr. W. Junk’s - Gravenhage, pp. 77–172.
10. Rehn, J.A.G. (1913) A contribution to the knowledge of the Orthoptera of Argentina. *Proceedings of the Academy of Natural Sciences of Philadelphia*, *65*, 273−379.
11. Rehn, J.A.G. (1920) Records and descriptions of Brazilian Orthoptera. *Proceedings of the Academy of Natural Sciences of Philadelphia*, 72, 214−293.
12. Rocha e Silva Albuquerque, I. 1964. Checklist dos Blattaria brasileiros. *Boletim do Museu Paraense Emilio Goeldi (Nova Serie) Zoologia* 41: 1–37.
13. Rocha e Silva Albuquerque, I. 1971. Sobre alguns Blattaria de Santa Catarina, Brasil (Dictyoptera). Revista Brasileira de Biologia 31: 329–335.
14. Rocha e Silva Albuquerque, I. 1972. Inventario dos Blattaria da Amazônia, com descrição de três espécies novas. Boletim do Museu Paraense Emilio Goeldi (n.s.) Zoologia 76:1-38.
15. Rocha e Silva Albuquerque, I. 1982. Lista dos Blattodea do Município do Rio de Janeiro, RJ, Brasil – (Dictyoptera). Boletim do Museu Nacional, Nova Serie, Zoologia 304: 1–20.
16. Rocha e Silva Albuquerque, I. & Vasconcelos, S. 1987. Nova contribuição ao conhecimento da fauna de Blattaria (Dictyoptera) do Alto da Mosela, Petrópolis, RJ, Brasil, com descrição de três espécies novas. Boletim museu nacional do Rio de Janeiro, Nova Série, Zool., Rio de Janeiro, 312:1-19.
17. Roth, L.M. (1970) Evolution and taxonomic significance of reproduction in Blattaria. *Annual Review of Entomology*, 15, 75–96.
18. Saussure, H. (1864a) *Orthopteres de L’Amerique Moyenne*. Mémoires pour servir à l'histoire naturelle du Mexique, Genève, 255 pp.
19. Saussure, H. (1864b) Blattarum novarum species aliquot. *Revue et Magasin Zoologie*, 2, 341−349.
20. Stâl, C. (1855) Entomologiska Notiser. *Ofversigt af Kongliga Vetenskaps-Academien förhandlingar*, 12, 342−355.
21. Thunberg, C.P. (1826) Blattarum novae species descriptae. *Mémoires de l'Académie impériale des sciences de St. Pétersbourg*, 10, 276 pp.
22. **Vanschuytbroeck, P.**1969. Catalogue des Blattariae conservés dans les collections entomologiques de l’Institut royal des Sciences naturelles de Belgique. Polyphagoidea et Blaberoidea. Bulletin of the Royal Belgian Institute of Natural Sciences, **45(14)**: 1-21
23. Walker, F. (1868) *Catalogue of the specimens of Blattarie in the collection of the British Museum*. British Museum, London, 239 pp.
