# Supplementary material for: The informative value of museum collections for ecology and conservation: A comparison with target sampling in the Brazilian Atlantic forest
Source: PLoS One. 2018 Nov 14;13(11):e0205710. doi: 10.1371/journal.pone.0205710 (PMC6235285; doi:10.1371/journal.pone.0205710)
Supplement: S1 Table — (DOCX) [file pone.0205710.s002.docx]

**S1 Table. List of Natural History Collections with specimens of *Monastria*.**

| Abbreviation | Institution | Locality |
| --- | --- | --- |
| MZUSP | Museu de Zoologia da Universidade de São Paulo | Sao Paulo, Brazil |
| MNRJ | Museu Nacional do Rio de Janeiro | Rio de Janeiro, Brazil |
| INPA | Instituto Nacional de Pesquisas da Amazônia | Manaus, Brazil |
| MNHN | Muséum National d’Histoire Naturelle | Paris, France |
| MHNG | Muséum de Histoire Naturelle de la Ville de Genève | Geneva, Switzerland |
| NRM | Swedish Museum of Natural History | Stockholm, Sweden |
| MZLU | Lund Museum of Zoology | Lund, Sweden |
| ME | Museum of Evolution | Uppsala, Sweden |
| ZFMK | Zoologisches Forschungsinstitut und Museum Alexander Koenig | Bonn, Germany |
| NHM | Natural History Museum | London, United Kingdom |
| IRSNB | Institut royal des Sciences naturelles de Belgique | Brussels, Belgium |
